# SNR improvement in GRE-EPI first-pass myocardial perfusion images with non-rigid body registration and KLT filtering

**DOI:** 10.1186/1532-429X-11-S1-P77

**Published:** 2009-01-28

**Authors:** Georgeta Mihai, Yu Ding, Hui Xue, Yiucho Chung, Jens Guehring, Orlando P Simonetti

**Affiliations:** 1 The Ohio State University, Columbus, OH USA; 2 Siemens Corporate Research, Princeton, NY USA; 3 Siemens Healthcare USA, Inc, Columbus, OH USA

**Keywords:** Perfusion Image, Parallel Imaging Technique, Detectable Artifact, Artifact Level, Prior Registration

## Introduction

Signal-to-noise ratio (SNR) and contrast-to-noise ratio (CNR) are often limited in first-pass myocardial perfusion images due to the demands for ultra-fast image acquisition and the use of parallel imaging techniques. Spatial or temporal low pass filtering can enhance SNR but may blur boundaries and generate artifacts. The Karhunen-Loeve Transform (KLT) filter takes advantage of temporal correlation to remove random noise without compromising either spatial or temporal resolution, thereby enhancing SNR in dynamic images [1, 2], but the performance of this filter has not been carefully evaluated for first-pass perfusion CMR. Image registration to compensate for respiration and variability in ECG triggering is a necessary step in quantitative evaluation of first-pass images. We hypothesize that registration can also increase the temporal correlation between images and reduce the potential for artifacts induced by KLT filtering.

## Purpose

The purpose of this study is to demonstrate that the combination of non-rigid registration and KLT filtering significantly improves CNR between normal and abnormally perfused myocardium without introducing blurring or other image artifacts.

## Methods

Ten consecutive first-pass perfusion datasets interpreted positive for ischemia or infarction were processed and analyzed retrospectively. All images were acquired using GRE-EPI with TSENSE acceleration rate 2 on a 1.5 T MR system (MAGNETOM Avanto, Siemens Healthcare, Germany). For each subject, all slices showing clinically interpreted perfusion defect were included in the analysis. Non-rigid body registration [3] was first performed on each series to allow for semi-quantitative analysis of signal enhancement, and to improve the correlation of dynamic images prior to KLT filtering [2]. The image frame showing peak enhancement in normal tissue was selected and regions of interest (ROI) were manually drawn in abnormal and normal myocardium for CNR calculation according to the equation CNR = (S_nornal_ - S_abnormal_)/σ_n_, where S_nornal_, S_abnormal_ are the mean signal intensities of normal and abnormal myocardium and σ_n_ is the standard deviation of the noise from a region outside the body. Identical ROI's were drawn on filtered (registration + KLT) and unfiltered images for evaluation of CNR improvement. In order to assess blurring or other artifacts induced by filtering, KLT filtered images both with and without prior non-rigid registration were evaluated by two experienced observers. Artifact level relative to the unfiltered image series was scored as: (1) none, (2) mild, (3) moderate and (4) severe.

## Results

**Figure 1:**
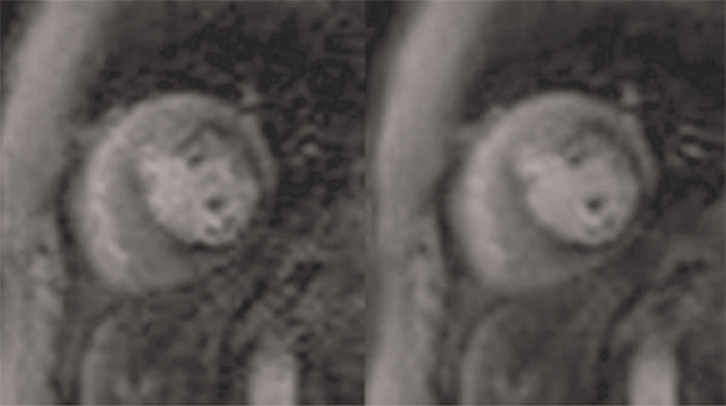
**First-pass perfusion image before (left) and after (right) KLT filtering**. KLT filter provides marked noise suppression without loss of contrast between normally and abnormally perfused regions.

The overall SNR of perfusion images increased with filtering. Figure 1 demonstrates the effect of the KLT filter on the registered perfusion images for one subject. There is a statistically significant increase in CNR with KLT filtering (mean 181.67 ± 27.75, p < 0.001), ranging from 129% to 216% increase in CNR after filtering. Prior non-rigid registration reduced the occurrence of filter induced artifacts. Mild artifacts were detectable in the images filtered without prior registration (mean score 1.65 ± 0.07) as opposed to almost no detectable artifacts induced by KLT filtering with registration (mean score 1.08 ± 0.08).

## Conclusion

The combination of non-rigid registration and KLT filtering was shown to increase the SNR of GRE-EPI perfusion images, with a direct increase of CNR between normal and abnormal regions. Subjective evaluation of image artifacts revealed no significant blurring or other artifacts caused by filtering, provided non-rigid registration was performed first.
